# Using the theory of planned behaviour to model antecedents of surgical checklist use: a cross-sectional study

**DOI:** 10.1186/s12913-015-1122-7

**Published:** 2015-10-07

**Authors:** Anna C. Mascherek, Katrin Gehring, Paula Bezzola, David L. B. Schwappach

**Affiliations:** Patient Safety Switzerland, Asylstrasse 77, 8032 Zurich, Switzerland; Institute of Social and Preventive Medicine (ISPM), University of Bern, Finkenhubelweg 11, 3012 Bern, Switzerland

**Keywords:** Surgical checklist implementation, Theory of planned behaviour, Group mean differences, Managerial function

## Abstract

**Background:**

Compliance with surgical checklist use remains an obstacle in the context of checklist implementation programs. The theory of planned behaviour was applied to analyse attitudes, perceived behaviour control, and norms as psychological antecedents of individuals’ intentions to use the checklist.

**Methods:**

A cross-sectional survey study with staff (*N* = 866) of 10 Swiss hospitals was conducted in German and French. Group mean differences between individuals with and without managerial function were computed. Structural equation modelling and confirmatory factor analysis was applied to investigate the structural relation between attitudes, perceived behaviour control, norms, and intentions.

**Results:**

Significant mean differences in favour of individuals with managerial function emerged for norms, perceived behavioural control, and intentions, but not for attitudes. Attitudes and perceived behavioural control had a significant direct effect on intentions whereas norms had not.

**Conclusions:**

Individuals with managerial function exhibit stronger perceived behavioural control, stronger norms, and stronger intentions. This could be applied in facilitating checklist implementation. The structural model of the theory of planned behaviour remains stable across groups, indicating a valid model to describe antecedents of intentions in the context of surgical checklist implementation.

**Electronic supplementary material:**

The online version of this article (doi:10.1186/s12913-015-1122-7) contains supplementary material, which is available to authorized users.

## Background

The World Health Organization’s (WHO) surgical checklist is an effective intervention to decrease morbidity and mortality in surgical procedures [1–4] and, thus, to increase patient safety. The checklist is now strongly recommended for adoption by international experts as an effective, yet economically simple intervention [5]. One important aspect in implementing the checklist has recently and repeatedly been stated: Simply implementing the checklist does not necessarily lead to improvements in patient safety [6]. The essential requirement for the checklist to be effective is staff compliance [1, 4]. However, compliance rates are often far below 100 % [7–9]. A multitude of possible reasons on organisational as well as individual level have been proposed to explain low compliance rates, for example lack of knowledge [10] and the way the checklist is implemented in the first place and how it is accompanied by supportive activities [11].

**Fig. 1:**
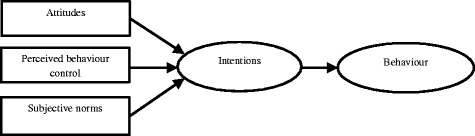
Conceptual model of the theory of planned behaviour [38]

Individual-centred explanations draw on psychological antecedents of intentions to either use or not to use the surgical checklist. A widely used theory to explain motivation of individuals to engage in health related behaviour is the "the theory of planned behaviour" (TPB) [12, 13]. To date, the TPB seems to be the most popular theoretical framework in order to explain determinants and antecedents of health-related behaviour. It has been used in different health behaviour contexts such as smoking, dieting, or exercising [12, 14–16]. More recently, the theory has been applied to engagement in patient safety-related behaviours, for example, patients’ involvement in error-preventing behaviours in Chemotherapy and infection prevention [17–19]. However, the TPB has not only been applied in the context of general health-related behaviour but also in terms of explaining facilitators and barriers of behaviour change of health-care workers concerning patient safety. The theoretical domains framework (TDF) tries to provide a theoretical framework to understand and explain behavioural change of individuals in the health-care domain [20]. Different theoretical approaches (e.g. TPB) have been combined in the TDF in order to explain, when and how and why individuals engage in patient safety behaviour. In a study by Taylor et al. [21] the TDF has been applied to explain the mechanisms and barriers underlying behavioural change concerning the positioning of nasogastric tubes prior to feeding. Aspects that added to the understanding were, amongst others, borrowed from the TPB. The TPB, hence, seems to be a reasonable construct to apply when trying to explain possible behaviour changes in health-care workers behaviour. In brief, the TPB states that intentions to perform a behaviour are influenced by three major factors: a) attitudes towards the behaviour, that is whether it is evaluated as favourable or unfavourable, b) subjective norms, that is the social pressure that is perceived concerning the performance or not-performance of the behaviour, and c) perceived behaviour control, that is to which extent individuals perceive themselves as being capable to successfully perform the behaviour [12]. All three components affect the formation of intentions to perform the behaviour or not (see Fig. 1). The TPB has also been applied to patient safety related behaviours: Schwappach and Wernli [17] studied the relationship between patients’ attitudes, norms, and perceived behaviour control, as well as patients’ intentions to contribute to drug administration safety during chemotherapy. They found that attitudes, norms, and perceived behaviour control significantly contributed to the patients’ intentions. Luszczynska and Gunson [19], for example, found a significant relation between perceived behaviour control and the intention to ask staff about hand washing in patients with MRSA. O’Boyle et al. found the TPB variables to predict health care workers’ intention to handwash, and their intention was related to self-reported hand hygiene [22]. Outside healthcare, Fogarty and Shaw used the TPB to model procedural violation behaviours in aircraft maintenance which are often associated with incidents and accidents [23]. Their model highlighted the importance of management attitudes and group norms as direct and indirect predictors of violation behaviour.

Generally, the more favourable the attitudes, the stronger subjective norms and the greater the perceived behavioural control, the more likely intentions in favour of the behaviour are built. Regarding the antecedents of intentions, Ajzen and Manstead [12] summarized different studies concerning a broad range of health-care related behaviour. In studies on physical exercise, the use of illicit drugs, eating a low-fat diet, consuming dietary products, and performing breast self-examinations, they found correlations between attitudes and intentions of .42 and .70, between subjective norms and intentions of .33 and 55, and between perceived behaviour control and intentions of .48 and 80. These studies indicate that all three components serve as a valuable predictor of intentions to perform a specific behaviour.

Intentions in turn are predictive of actual behaviour, though the strength of this relationship varies over situations and is influenced by different other aspects [12, 24]. It has been argued that intentions and actual behaviour can only be consistent if beliefs are the same across both, the hypothetical and the real situation. Although perceived behaviour control, attitudes and norms contain a stable core, that is, invariant across situations, they are still influenced by different contextual cues. Depending on the situation, different aspects of perceived behaviour control and attitudes become more salient than others and may, hence, determine the actual behaviour. Hypothetical and actual situations are qualitatively different and trigger different facets of attitudes, norms, and perceived behaviour control. The more closely related perceived behaviour control and attitudes are to the real situation, the greater the predictive value of the TPB [24]. That is the closer attitudes and perceived behaviour control contentually resemble the real situation, the greater the predictive power. If individuals voice attitudes and perceived behaviour control that do not closely match the situation under study, the predictive value of this self-reporting decreases. A meta-analysis of the intention-behaviour relationship suggests that a medium-to-large change in intention is needed to achieve a small-to-medium change in behaviour. Behaviours are performed in “social context” and intentions usually exert less strong influence when there is potential for social reaction [25]. However, concerning intention-building, the TPB has proven to be of predictive value [12].

To the best of our knowledge, the TPB has not been applied to the context of determinants of surgical checklist use. With checklist use as behaviour of interest, the TPB would predict that positive beliefs about the usefulness of the checklist and its value for patient safety would positively influence intentions to use the surgical checklist. If individuals perceive strong subjective norms that their co-workers and/or superiors expect the use of the checklist and individuals highly value these expectations, intentions to use the checklist should be positively influenced. Finally, if individuals perceive high levels of control over the target behaviour to be successfully conducted, intentions to use the checklist should be positively influenced. However, use of the checklist is embedded in a highly social context. It requires teamwork with co-workers of different professions, roles, hierarchies and power differentials. Intentions to use the checklist may therefore be influenced by professional relations and power. Attitudes, norms, and perceived behaviour control may also be systematically influenced by hierarchical positions. Individuals holding a leading position within a team usually have greater decisional power than individuals without an executive position. Perceived behavioural control describes peoples’ perception of the difficulty to successfully perform the behaviour of interest. Although perceived behavioural control reflects a person’s perception of control and not his or her objective level of control, the experience of being able to make decisions which influence team routines may support a general feeling of greater behavioural control in a specific environment. Hence, perceived behaviour control might be more distinct in individuals with managerial function. Due to greater autonomy of decision that is an inherent aspect of leadership positions, the perceived capability of successfully conducting a specific behaviour should be greater. As a consequence, levels of perceived behaviour control should be higher which should result in stronger intentions. We would hence expect perceived behaviour control as being more distinct in individuals with managerial function and, ultimately, being of greater predictive power concerning intentions. Compliance with checklist use has often been found to vary [1, 26, 27]. However, reasons for the variation are difficult to find. In analysing whether the general TPB model fits the data of the present study, we present an important prerequisite for future studies to analyse the relation of behaviour antecedents as described with the TPB and actual behaviour.

Taken together, the aims of the present study were twofold: First, we used the TPB to model the relationship between norms, attitudes and perceived behavioural control on intentions to use the WHO surgical checklist. Second, we examined whether individuals with and without managerial function differ systematically with respect to the variables of the TPB.

## Methods

### Design

A cross-sectional study was conducted by the Swiss Patient Safety Foundation in October/November 2013 in the context of a larger implementation intervention in 10 Swiss hospitals. The 10 hospitals participated in a project to implement the comprehensive use of the WHO-surgical checklist. Hospitals were recruited via a national open tender. All Swiss hospitals could apply for participation as a reaction of the open tender. Participation was promoted as prestigious. Hospitals applied for the participation in the checklist implementation program with automatic participation in the evaluation program of which the data for the present study originates. Financial compensation was not offered whatsoever. In total, 32 hospitals applied for participation from all over Switzerland. 10 out of the 32 applicants were selected for participation in the implementation program. Criteria for hospital selection were established in order for the selected hospitals to be as representative as possible, however, still meet certain criteria necessary for participation in the implementation program. Criteria for hospital selection were hospital size, hospital speciality, geographical region within Switzerland, explicit commitment from senior surgeons and anaesthetists, and whether or not they had already implemented some kind of checklist into their operating room–routine (OR). The questionnaire was part of a larger data-collection-episode in which two separate questionnaires (one covering the TPB items and one related to safety climate, not reported herein, see Gehring et al. for details [28]) were used. A print version of the questionnaire was sent to the hospitals and locally distributed. The questionnaire was developed in German and translated into French by professional translators. The survey sample consisted of all members of the Operating Room (OR) teams of the respective hospitals (doctors, nurses, scrub nurses, surgical technicians, and attendants for surgical positioning). Subjects were invited to participate by the hospitals’ project teams and repeatedly reminded to participate throughout the data-collecting period. This study was conducted as part of a quality improvement project. The study design and the data collection did not require approval of an ethical committee in Switzerland referring to Article 1 and 2 of the Federal Act on Research involving Human Beings (Human Research Act, HRA) [29].

### Survey

The survey was developed to assess use of, knowledge of and attitudes towards the WHO-surgical checklist. It was developed on the basis of extensive review of the literature and piloted and validated in a previous study [30]. The survey consisted of three conceptual parts. The first part asked questions related to checklist usage. In the second part subjective and objective knowledge about the WHO-checklist was assessed. In the third part, TPB constructs, i.e., attitudes, norms and perceived behaviour control towards checklist use as well as intentions to use the checklist were assessed [12, 13]. Perceived behaviour control (e.g.” I have a say on the correct checklist use within my team”) and norms (e.g. “Surgeons look down upon checklist use”) were measured with 4 items each, attitudes (e.g. “Checklist use enhances paying attention to patient safety”) wit 7 items, and intentions with  (e.g. “It is my plan to carefully mind the use of the checklist”) with 6 items, each rated on a 7-point-Likert-scale ranging from “do not agree at all” to “completely agree” (for the wording of all TPB-items see Table 1). High scores indicate a positive evaluation of the checklist. Four items had to be reverse coded for data analyses. The survey was pre-tested with individuals from all professions and languages. Managerial function was assessed with a single item dichotomously coded as “yes, holding a managerial function” and “no, not holding a managerial function”.

**Table 1:** Wording, respective factor and mean score per group of the TPB items (items translated from the German original)

Item no.	Item (Factor)	Mean MF(SD)	Mean w/o MF (SD)
V03	Within my daily routine I can apply the checklist correctly. (pbc)	5.6 (1.5)	5.3 (1.6)
V05	I promote checklist use amongst my team-members. (pbc)	6.0 (1.4)	5.4 (1.7)
V17	My behaviour influences whether or not the checklist is used correctly at a given surgical procedure. (pbc)	6.4 (1.0)	6.1 (1.3)
V21	I have a say on the correct checklist use within my team. (pbc)	5.9 (1.5)	5.2 (1.7)
V04	Checklist use facilitates speaking up in the OR. (att)	5.8 (1.4)	5.7 (1.4)
V06	Checklist use is far too time-consuming. (att)	5.7 (1.6)	5.7 (1.5)
V09	Checklist use decreases adverse events. (att)	6.3 (1.1)	6.1 (1.2)
V13	Checklist use hinders the flow of information among OR-team members. (att)	6.1 (1.6)	6.0 (1.5)
V19	Checklist use enhances paying attention to patient safety. (att)	6.5 (0.8)	6.4 (0.9)
V20^a^	Checklist use interferes with my tasks. (att)	5.9 (1.6)	5.7 (1.6)
V22^a^	A well-functioning OR-team does not need a checklist. (att)	6.4 (1.3)	6.4 (1.1)
V15	Surgeons look down upon checklist use. (norms)	4.5 (1.9)	4.4 (1.9)
V18	My colleagues take checklist use serious. (norms)	5.7 (1.3)	5.6 (1.3)
V23	My supervisor promotes checklist use. (norms)	6.3 (1.3)	6.1 (1.3)
V24	I am expected to use the checklist seriously. (norms)	6.4 (1.2)	6.1 (1.4)
V07	I will promote checklist use. (int)	6.3 (1.0)	5.8 (1.3)
V08	I will support my colleagues with using the checkliste. (int)	6.5 (0.9)	6.1 (1.2)
V10	Next time I am up to decide, I will apply the checklist. (int)	6.2 (1.3)	6.2 (1.1)
V11	It is my plan to carefully mind the use of the checklist. (int)	6.4 (1.0)	6.2 (1.0)
V16	I want the checklist to be used with every patient. (int)	6.6 (0.9)	6.5 (0.9)
V25^b^	It is my duty to correctly use the checklist. (int)	6.6 (0.9)	6.3 (1.2)

*MF* managerial function, *w/o MF* without managerial function, *SD* standard deviation, *pbc* perceived behaviour control, *att* Attitude, *int* intentions

^a^items were allowed to load on attitude instead of perceived behaviour control and norms, respectively, after inspection of the confirmatory factor model

^b^= item was allowed to load on intentions instead of norms after inspection of the confirmatory factor model

### Data analyses

Individuals who answered less than 60 % of the relevant items of the questionnaire were dropped from analysis. In a first step, confirmatory factor models were fitted to the data to test the measurement model and verify the theoretical structure assumed. In a second step, structural equation modelling was used to test for group differences between individuals with and without managerial function. Analysis of measurement invariance was conducted as a prerequisite for analysing differences on latent level to provide a valid basis for examining group differences on the latent level. Measurement invariance ensures that differences can be ascribed to differences between groups that are not due to different functioning of the measure per se (for details on estimating measurement invariance in the present study, see Additional file 1).

We report the Comparative Fit Index (CFI) and the Root Mean Square Error of Approximation (RMSEA) as measures for model fit. Values of the CFI above 0.90 denote a well-fitting model, whereas for the RMSEA values less than 0.08 may be interpreted as acceptable model fit [31]. We used maximum likelihood with missing values estimation (mlmv) for our analyses, implying missing values to be missing at random [32]. Group differences between individuals with and without managerial function were examined by T-tests. All analyses were conducted using STATA v13.1 [33].

## Results

### Sample

1139 of the 2083 invited Health Care Professionals (HCPs) completed the survey (54.7 % response rate). 191 individuals were excluded from the analysis, because they answered less than 60 % of the relevant items. Additional 82 were excluded due to invalid answers (e.g., individuals who ticked off several boxes per item). This resulted in 866 individuals included into the analyses of the present study. Sample characteristics are presented in Table 2 for managerial function separately.

**Table 2:** Sample characteristics by managerial function. (data not adding up to 100 % are due to missing values)

		Total (*N* = 866)	Without managerial function	With managerial function
			*n* = 582	*n* = 284
Survey language	German	77.7	81.3	70.4
	French	22.3	18.7	29.6
Gender	female	49.2	58.1	30.6
	male	49.1	40.1	67.3
Mean age in years (SD)		40.9 (10.4)	37.3 (10.0)	45.3 (9.0)
Education	Doctors	57.7	53.3	67
	Theatre nurses	19.3	20.8	16.2
	Surgical technicians	16.2	17.7	13
	Attendants for surgical positioning	4.5	5.5	2.5
	Others	1.3	1.6	.7
Managerial function	Yes	32.8		
	No	67.2		
Years of professional experience	0 - 2 years	19.4	25.1	7.8
	2 - 5 years	18.6	20.6	14.4
	5 - 10 years	20.6	21.7	18.3
	10 - 20 years	21.9	17.5	31
	more than 20 years	18.7	14.4	27.5
hours spent in the OR in an average week	None	2.4	2.8	1.8
	0 to 8	16.8	17.4	15.6
	8 to 16	18.9	18.5	19.9
	16 to 24	15.7	14.2	18.8
	24 to 32	14.9	13.1	18.4
	32 to 40	17.0	19.2	12.4
	more than 40	13.7	14.5	12.1

### Model estimation

**Fig. 2:**
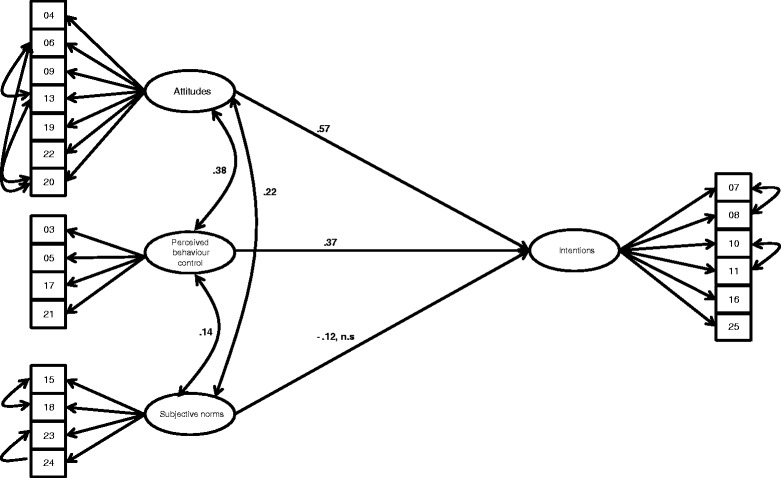
Final model of the theory of planned behaviour in the context of surgical checklist use. (CFI = 0.89, RMSEA = 0.076). Note: Effects of attitudes, perceived behavioural control, and norms on intentions to use the surgical checklist. Observed manifest variables (survey items) are presented as rectangles. Latent variables are presented as ellipses. Path coefficients and covariances on latent level were constrained to be equal across groups as this did not lead to a significant decrease in model fit. n.s. = not significantly different from zero

In a first step the confirmatory factor model was estimated with the respective items loading on the theoretically assumed corresponding factor. This first model did not fit the data well (CFI = 0.86, RMSEA = 0.087). Based on inspection of the modification indices and theoretical considerations, it seemed reasonable for a few items to load on a different factor than on the one initially specified. Hence, in a second model, 3 items were allowed to load on a different factor. Namely, item 22 was allowed to load on attitudes instead of norms, item 20 was also allowed to load on attitude instead of perceived behaviour control, and item 25 was allowed to load on intentions instead on norms. Additionally, covariances between measurement errors were allowed within factors. This second model fitted the data well (CFI = 0.89, RMSEA = 0.076). Hence, we accepted model 2 as our basic TPB-model (see Fig. 2). Cronbach’s alpha indicated satisfactory internal consistency of the final scales (norms *α* = .72, attitudes, *α* = .75, perceived behaviour control, *α* = .76, and intentions *α* = .91). Based on the final model we conducted multiple group comparisons in order to test for differences in the structural model between individuals with managerial function and individuals without. Strong measurement invariance was found to hold (see Additional file 1 for details). Structural coefficients and covariances between norms, attitudes, perceived behaviour control, and intentions were constrained to be equal for both groups. This did not lead to a significant decrease in model fit, indicating that path coefficients and covariances did not differ significantly between groups (CFI = .89, RMSEA = .076). Hence, we accepted model 2 as our final model with constraining latent path coefficients and covariances to be equal across groups. Intentions were most strongly influenced by attitudes (*β* = .57, *p* < 0.001). Perceived behaviour control also significantly influenced intentions (*β* = .37, *p* < 0.001), however, norms did not significantly influence intentions (*β* = −.12, n.s.). Covariances between norms and perceived behaviour control, norms and attitude and attitude and perceived behaviour control were significant on *p* < .001 and are depicted in Fig. 2.

### Group differences

Accepting the changes concerning item loadings on specific factors that were revealed by the CFA, group differences were analysed with respect to the changed composition of the scale. Norms, attitudes, and perceived behaviour control differed significantly between individuals with managerial function and individuals without. Group differences in responses to the items of the four scales are depicted in Table 1. Differences on item level are consistent with the direction of differences on scale level. On scale level, individuals with managerial function scored significantly higher on norms (*t*_(864)_ = 2.75, *p* <. 0.05.; *M* = 5.6, *SD* = 1.1 for individuals without managerial function, *M* = 5.9, *SD* = 1.0 for individuals with managerial function), perceived behaviour control (*t*_(864)_ = 5.72, *p* < 0.05.; *M* = 5.6, *SD* = 1.2 for individuals without managerial function, *M* = 6.1, *SD* = 1.0 for individuals with managerial function), and intentions (*t*_(864)_ = 3.77, *p* < 0.05.; *M* = 6.2, *SD* = 1.0 for individuals without managerial function, *M* = 6.5, *SD* = 0.8 for individuals with managerial function). Differences in attitudes between groups did not reach significance (*t*_(864)_ = 1.5, n.s.; *M* = 6.0, *SD* = 0.9 for individuals without managerial function, *M* = 6.1, *SD* = 1.0 for individuals with managerial function).

## Discussion

In the present study, we assessed attitudes, norms, perceived behaviour control and intentions to use the surgical safety checklist among OR staff from 10 Swiss hospitals and applied the TPB to model the antecedents of intentions to use the checklist. Generally, norms, attitudes, perceived behaviour control, and intentions were rated highly, indicating a positive evaluation of the checklist as a tool to improve patient safety in surgery. Regarding norms, perceived behaviour control, and intentions, individuals with a managerial function scored significantly higher than individuals without. Mean differences in attitudes pointed into the same direction, however, did not reach significance. These results indicate that individuals with a managerial function are attuned more positive to the checklist in general than individuals without managerial function. Differences in the evaluation of cultural factors within an organisation alongside hierarchical functions are known from the literature. Studies on safety climate, for example, repeatedly report more positive ratings from individuals in higher hierarchical positions [34]. The more positive evaluation of checklist use might reflect the fact that individuals with managerial function carry responsibility to a greater extent. Hence, they might stronger identify with recommended interventions to enhance patient safety. Additionally, because they might be less confronted with practical obstacles to implement the checklist as a behavioural routine into OR-staffs daily routine, one might conclude that individuals with managerial functions view checklist use from a more idealistic, less practical point of view. Evaluating checklist use on a more abstract level without strongly considering possible practical obstacles might consequently lead to a more positive basic attitude. However, from the data of the present study, this explanation remains speculative and should be objective of future studies. The generally more positive basic attitude of individuals with managerial function could be positively used in implementation programs as such that they may be appointed to role model positions when it comes to actual surgical checklist implementation. This is true for establishing norms, as individuals with managerial function might act as “significant opinion leaders” within the work-context. While perceived behaviour control might simply be a function of greater freedom of decision inherent in higher positions, norms are idiosyncratic and might, hence, be influenced on individual level.

Looking on item-level for perceived behavioural control, individuals with managerial function clearly felt that they had greater influence on checklist use. Moreover, they considered themselves as actively promoting the checklist more than individuals without managerial function did. Not surprisingly, individuals with managerial function felt that their sphere of influence, concerning whether the checklist is used or not, was greater. Taking a closer look at attitudes, individuals with managerial function reported higher levels of agreements with items that generally describe the checklist as a useful tool to enhance patient safety. Awareness of general patient safety issues on system-level might, hence, be greater in individuals with executive power. Taken together, the generally positive attitudes and the greater actual control over the target behaviour might pioneer and facilitate checklist implementation. Raising awareness for patient safety issues in general and establishing norms via role models might additionally contribute. In quality improvement programs, the importance of role-models (so-called “champions”) for successful implementation has long been recognized [35]. Champions are role-models who hold high impact-positions within a hospital or ward. Champions are expected to actively promote the target behaviour themselves and to intervene if target behaviour is not shown by others around them. The differences on item-level show, that individuals with managerial function might be well suited to act as champions in a checklist implementation program as they, on an average level, value the checklist higher and feel more able to implement it.

Concerning our model, the TPB model fitted the data of the present study reasonably well. The structural part, that is the part describing the relation between the target variables, did not significantly differ between individuals with managerial function and individuals without. This indicates that differences between the groups are present concerning the level, however, not the general structure of the relation between the variables. This is an important finding. The TPB can thus be seen as a stable and valid construct to describe interdependencies between psychological determinants of intentions in the applied context of surgical checklist implementation. However, we also found some counterintuitive results. In particular, norms did not significantly contribute to the explanation of intentions in our data. A weak or non-significant relation between norms and intentions has been reported in the literature (cf. Armitage & Conner, 2001). It has been argued that intentions are stronger influenced by the more personal factors attitude and perceived behaviour control. Also, it has been proposed that the conceptualisation of the construct “norms” might be too narrow to exhibit significant influence on intention-building as substantial aspects of norms might not have been captured [36, 37]. In the context of surgical checklist implementation we present an additional explanation that, however, is in line with the idea of norms being conceptualised “too narrow”. Since the WHO launched the surgical checklist in 2009 worldwide with an accompanying study on its effectiveness, numerous studies have proven the effectiveness of the surgical checklist to enhance patient safety since then. Hence, we reason that surgical checklist use as a normative standard might be as established that it does not add to the understanding of varying intentions anymore. The normative standard might be generally established and, hence, might exist independently of intentions to actually use it or not. Individual attitudes and perceived behaviour control seem to exert stronger influence on intentions than generally established norms to use it. In line with this argumentation is the distinction between injunctive norms (i.e., what significant others think an individual should do) and descriptive norms (i.e., what significant others really do) [37]. While using the surgical checklist might be strongly established on an injunctive norm-level, this could be different on descriptive-norm level. Checklist use as a general norm describing the “gold standard” and, accordingly, the notion of what people *should do* might be well established in Swiss hospitals. However, the actual checklist use within a daily routine, that is what co-workers actually *do*, might be different. In the present study, we did not differentiate between those two norm aspects; hence, they cannot be accounted for separately, which could explain the non-significant contribution of the factor norms. Future studies should focus on the differentiation of injunctive and descriptive norms already concerning the operationalization on item level to further elaborate on this relation.

### Limitations

The study has some limitations that have to be taken into account. The first aspect concerns sample selection. Because the sample was part of a larger quality improvement project, sample size with respect to the present study was not calculated in advance. However, a post-hoc power test found a power of 86 to detect a small effect in the present sample size. As a power of 80 is usually considered as satisfactory, the sample size can be considered as large enough for the present study. Only staff from hospitals participating in the checklist implementation project was sampled. Although the 10 hospitals were selected in order to cover a broad range of health care institutions in Switzerland the sample is not representative and thus, the generalizability of our findings is unclear. Also, because data were assessed within the implementation project, participants are likely to represent a relatively well informed group. In line with that, we cannot rule out bias from selective non-response as we have no data about non-responders. This, again, limits the generalizability of our results. Also, managerial function was assessed with a single self-rating item. No formal definition was given to study participants on when to rate their own position as “managerial”. Though the term “managerial function” is often used in Swiss hospital care and we expect a common meaning of this terminology, we cannot rule out bias in the reports of managerial function. Another limitation that has to be discussed is the unassessed relation between intentions and actual behaviour. In the present study we did not include actual behaviour into our analyses, but rather focussed on the antecedents. Behaviours are performed in “social context” and when there is potential for social reaction they are usually less strongly determined by intentions [24]. Especially within the very structured context of a hospital, social context variables and regulations that are beyond the influence of an individual, might significantly influence and ultimately change the actual behaviour compared to original intentions. Social context could hinder intentions to consistently lead to the related behaviour. This question cannot be answered from the data of the present study, however, remains an important aspect that should be addressed in future studies to explain the link between intentions and actual behaviour within the context of checklist implementation.

## Conclusion

We conclude that the TPB can be applied to the context of surgical checklist implementation in order to explain intentions. Individuals with managerial function exhibit higher levels, indicating a generally more positive tenor than individuals without managerial function. The structure between the TPB-variables remains stable between groups hence, the TPB can be applied independent of hierarchical positions in the context of surgical checklist use.

## Additional file

Additional file 1:
**Testing for measurement invariance.** (DOCX 20 kb)
