# Supplementary material for: Using the theory of planned behaviour to model antecedents of surgical checklist use: a cross-sectional study
Source: BMC Health Serv Res. 2015 Oct 7;15:462. doi: 10.1186/s12913-015-1122-7 (PMC4596358; doi:10.1186/s12913-015-1122-7)
Supplement: Additional file 1: — Testing for measurement invariance. (DOCX 20 kb) [file 12913_2015_1122_MOESM1_ESM.docx]

**Additional file 1: Testing for measurement invariance**

Structural equation modelling started with testing different degrees of measurement invariance. Without establishing measurement invariance, emerging differences could also be due to differences on manifest level, hence, causing difficulties in interpretation. According to Meredith and Horn [1], three degrees of measurement invariance were examined by constraining different parameters to be equal across the two groups: configural invariance, weak factorial invariance, and strong factorial invariance.

Scaling of the configural invariance model was achieved by fixing the factor variances to one and the factor means to zero.

The absolute goodness-of-fit of models was evaluated using the χ^2^-test and two additional criteria, the Comparative Fit Index (CFI) and the Root Mean Square Error of Approximation (RMSEA). Values of the CFI above .90 are considered to be adequate, whereas for the RMSEA values less than .08 indicate an acceptable model fit [2]. For assessing the relative fit of nested models, we used the χ^2^-difference test.

In the configural invariance model, each item loaded on the same assigned factor across groups. This model achieved an acceptable fit. Although the model exhibited a significant decrease in model fit concerning the χ^2^value, we accepted the model. χ^2^-values are sample size sensitive. With a sample size of 866 individuals, a significant χ^2^-value is acceptable, considering the fact the CFI and the RMSEA are within an acceptable range.

Subsequently, weak measurement invariance was tested for, requiring the factor loadings to be constrained to be equal across groups. As can be seen from Table 1, this led to a significant reduction in model fit in terms of *χ²* -differences compared to the model of configural invariance. The RMSEA improved slightly. The CFI basically remained stable. According to Cheung and Rensvold [3] the CFI and RMSEA still indicate an acceptable fit (CFI = 0.899, RMSEA = 0.075). We therefore accepted weak measurement invariance, as sample size was large and χ²-values become more sensitive for any deviation with increasing sample size.

In a last step, strong measurement invariance was tested for, requiring the intercepts of the measurement model to be equal across groups. This again led to a significant decrease in model fit in terms of χ^2^-differences. CFI and RMSEA remained stable as before. Additionally, inspection revealed a general “background noise” as a source of the loss of fit. There were no specific parameters accounting for the loss of fit. Rather, the model as a whole represented the data relatively worse. This seems reasonable as the model implies stronger constraints on the data set. Consequently, we accepted strong measurement invariance to hold across groups.

Table 1: Sequence of estimated measurement invariance models

| Model | χ^2^ | df | Δχ^2^ | Δdf | CFI | RMSEA | RMSEA 90% CI |
| --- | --- | --- | --- | --- | --- | --- | --- |
| Configural invariance | 1227.578* | 350 |  |  | 0.903 | 0.076 | 0.071-0.081 |
| Weak MI | 1281.426* | 371 | 53.848* | 21 | 0.899 | 0.075 | 0.071-0.080 |
| Strong MI | 1378.194* | 392 | 96.768* | 21 | 0.890 | 0.076 | 0.072-0.081 |

*Note:* df = degrees of freedom, CFI = Comparative Fit Index, RMSEA = root mean square error of approximation, CI = confidence interval, MI = measurement invariance

*N* = 866

* *p* < 0.05

References

1. Meredith W, Horn JL: **The role of factorial invariance in modeling growth and change.** In *New methods for the analysis of change*. Edited by Collins LM, Sayer AG. Washington DC: American Psychological Association; 2001:203-240.

2. MacCallum RC, Browne MW, Sugawara HM: **Power analysis and determination of sample size for covariance structure modeling.** *Psychological Methods* 1996, **1:** 130-149.

3. Cheung GW, Rensvold RB: **Evaluating goodness-of-fit indexes for testing measurement invariance.** *Structural Equation Modeling* 2002, **9:** 233-255.
